# The processing of visual food cues during bitter aftertaste perception in females with high vs. low disgust propensity: an fMRI study

**DOI:** 10.1007/s11682-021-00455-2

**Published:** 2021-02-16

**Authors:** Anne Schienle, Albert Wabnegger

**Affiliations:** Institute of Psychology, University of Graz, BioTechMedGraz, Universitätsplatz 2/DG, A - 8010 Graz, Austria

**Keywords:** Bitter perception, Visual food cues, Disgust propensity, fMRI

## Abstract

An extremely bitter taste can signal food spoilage, and therefore typically elicits disgust. The present cross-modal functional magnetic resonance imaging experiment investigated whether the personality trait ‘disgust propensity’ (DP; temporally stable tendency to experience disgust across different situations) has an influence on the processing of visual food cues during bitter aftertaste perception. Thirty females with high DP and 30 females with low DP viewed images depicting sweet food (e.g., cakes, ice cream) and vegetables, once in combination with an extremely bitter aftertaste (concentrated wormwood tea), and once with a neutral taste (water). Females highly prone to disgust (compared to low disgust-prone females) showed increased activity in the anterior cingulate cortex (ACC) and increased mPFC-insula connectivity when presented with the mismatch of a bitter aftertaste and visual cues of sweet food. The ACC is involved in conflict monitoring and is strongly interconnected with insular areas. This connection plays a critical role in awareness of changes in homeostatic states. Our findings indicate that the personality trait DP is associated with cross-modal integration processes of disgust-relevant information. Females high in DP were more alert to food-related sensory mismatch (pleasant visual features, aversive taste) than females low in DP.

**Supplementary Information:**

The online version contains supplementary material available at 10.1007/s11682-021-00455-2.

## Introduction

What is the function of disgust? From an evolutionary psychology perspective, the emotion of disgust evolved to protect humans from pathogen-transmitted diseases (e.g., Curtis et al. 2011; Tybur et al. 2009). Disgust motivates individuals to avoid, reject and/or remove pathogens (Oaten et al. 2009). Some researchers (e.g., Rozin et al. 2009) have argued that the most primitive or basic form of disgust (‘core disgust’) is built upon the distaste response, which is part of a preadapted bitter (toxin) avoidance system (Curtis et al. 2011). Spoiled or toxic food often tastes bitter (Glendinning 1994). The perception of a bitter taste, especially of high intensity, typically elicits disgust, which functions as a danger signal that the likelihood of contagion is high. Subsequently, oral rejection is initiated (gape with tongue extension, nausea) to free the body from these health-threatening substances. The typical facial expression of disgust may be a vestige of this rejection impulse (Rozin et al. 2009).

Personality research has observed marked individual differences in the extent to which disgust is experienced, suggesting that there may be different thresholds for the activation of the disgust-related disease avoidance mechanism (e.g., Olatunji et al. 2017). A relevant trait within this context is disgust propensity (DP). DP is the temporally stable tendency of a person to experience disgust across different situations (e.g., Schienle et al. 2002a, b). Additionally, bitter sensitivity (the ability to detect bitter compounds in food) is a temporally stable characteristic of a person (Herz 2011). Rozin et al. (2009) proposed a ‘bitter-disgust link’, suggesting a positive association between disgust propensity and bitter sensitivity. In line with this assumption, previous research has shown that individuals who were very sensitive to the bitter compounds 6-n-propylthiouracil (PROP) and quinine hydrochloride scored higher on DP questionnaires than individuals with low bitter sensitivity (Herz 2011; Herbert et al. 2014; Schienle and Schlintl 2019). These studies underline the close connection between bitter taste perception and DP.

At the neural level, bitter taste perception is associated with activation of specific regions in the insula and in the frontal/ parietal operculum, which are components of the gustatory cortex (e.g., Chikazoe et al. 2019). Several meta-analyses have identified general taste-sensitive brain regions in the insula, the orbitofrontal cortex (OFC), and the basal ganglia (BG; e.g., Veldhuizen et al. 2011; Yeung et al. 2017). The very same regions (insula, OFC, BG) are recruited when feelings of disgust are elicited by stimuli of different sensory modalities (e.g., visual or olfactory disgust stimuli; Schienle et al. 2002a; Wicker et al. 2003; for a brief review see Curtis et al. 2011). In particular, the insula has been identified as a central node of the neural disgust system (Wicker et al. 2003). The insular cortex integrates sensory information and is part of a central circuit subserving evaluative and affective processes (Murphy et al. 2003). The information exchange (functional connectivity) between the insula and other brain regions (e.g., medial prefrontal cortex (mPFC), OFC) shapes an individual’s experience of revulsion (e.g., Schienle et al. 2014).

Finally, the OFC, BG, mPFC, and insula are also involved in the processing of food-related information. For example, the viewing of images depicting appetizing food has been consistently accompanied by activation in the mentioned brain regions (e.g., Blechert et al. 2016; Frank et al. 2013).

In the present functional magnetic resonance imaging (fMRI) study, we investigated the neural underpinnings of the ‘disgust-bitter link’ in the context of food cue processing. We used a previously tested design (Wabnegger et al. 2018). In that study, the participants were presented with images depicting appetizing food (sweet food and vegetables), once in combination with a bitter aftertaste and once with a neutral taste (water). In the bitter condition, participants rinsed their mouths with highly concentrated wormwood tea which elicited a long-lasting bitter aftertaste. This intervention reduced the reported appetite for sweet food and enhanced the activity in the prefrontal cortex (Wabnegger et al. 2018).

The aforementioned aftertaste procedure was used in the present fMRI study to compare females with high vs. low DP. The sample was restricted to female participants since females typically report higher DP than men (e.g., Schienle et al. 2002b). Based on findings of individual variation in bitter sensitivity associated with DP (e.g., Herz 2011; Herbert et al. 2014; Schienle and Schlintl 2019), it was predicted that high disgust-prone females would rate the wormwood tea as more bitter and disgusting, compared to low disgust-prone females. Additionally, the DP_high group should show a more pronounced reduction of the appetitive value of sweet food cues that are coupled with a bitter taste than the DP_low group. Because bitter taste is an alarm signal of potential food spoilage (and disease risk) disgust-prone participants should report lower appetite ratings and higher arousal ratings during the combined processing of pleasant food images and bitter taste.

The analysis of the fMRI data followed a region of interest (ROI) approach. We analyzed brain activation in those ROIs that have been associated with the processing of pleasant visual food cues and unpleasant chemosensory stimuli, such as the insula, operculum, OFC, BG, and mPFC (Wicker et al. 2003; Chikazoe et al. 2019; Frank et al. 2013). Additionally, functional connectivity between the analyzed ROIs was investigated and compared between the two DP groups. Because the role of personality traits in cross-modal processing has not been covered in previous research, the fMRI analysis of the current investigation was exploratory and focused on possible changes in ROI activity and connectivity during the chosen gustatory-visual stimulation.

## Method

### Participants

A total of 60 normal-weight females (mean age: 24.3 years, SD = 6.1; mean years of education: M = 12.75, SD = 1.37) participated in the study; 87% were university students and 13% white-collar workers**.** All participants had normal or corrected-to-normal vision and reported having no history of psychiatric or neurological diagnoses, brain injuries, or substance abuse.

All participants provided written informed consent after a full explanation of the testing procedure. The study was conducted following the Declaration of Helsinki and had been approved by the ethics committee of the University.

### Measures

The participants completed the Questionnaire for the Assessment of Disgust Propensity (QADP; Schienle et al. 2002b). The QADP describes 37 situations (e.g., You touch the toilet seat with part of your body in a public restroom; you smell vomit), which have to be judged on 5-point scales concerning experienced disgust (0 = not disgusting; 4 = very disgusting). The QADP had a Cronbach’s alpha of .94 in the present sample. The mean QADP scores showed a bimodal distribution in the total sample (see supplementary Fig. S1). Therefore, we conducted a median split analysis and compared participants with high vs. low mean QADP scores (M_low = 1.54, SD = 0.32; M_high = 2.86, SD = 0.31). The two groups did not differ in mean age, years of education, and BMI (all *p* > .30).

The gustatory function was assessed using a standardized gustatory test (according to Landis et al. 2009). The test included four stimuli that were presented to the blindfolded participants with the following concentrations: sucrose: 0.4 g/ml, sodium chloride: 0.25 g/ml, quinine hydrochloride: 0.006 g/ml, citric acid: 0.3 g/ml. Distilled water was used as a solvent. Before the administration of each gustatory stimulus, the mouth was rinsed with water. The participants labeled the taste based on a list with four descriptors, i.e., sweet, sour, salty, and bitter (multiple forced-choice), and rated the perceived intensity (0–100%).

### Procedure

The study consisted of three sessions. The first diagnostic session included the questionnaire assessment and the standardized taste test. Afterward, the participants were invited to two fMRI sessions (separated by approximately 1 week). The fMRI sessions were preceded by an overnight fast and started with a rating of the current hunger level (1 = low; 9 = high).

- **Fluids:** The participants received 20 ml concentrated wormwood tea (with the bitter compound absinthin) in one condition of the experiment and 20 ml tap water in another condition. The tea was made with 10 teaspoons of dried herbal powder per 100 ml of water. The tea steeped for exactly 7 min and then cooled down to room temperature. Directly before the fMRI recording, the fluids were administered and held in the mouth for 30 s and then expectorated. A previous study had shown that the wormwood aftertaste continued to be present for at least 10 min (the time of picture presentation; see Wabnegger et al. 2018). The sequence of the conditions was counterbalanced. The aftertaste was rated according to perceived bitterness and disgust on 9-point Likert scales (1 = low, 9 = very high), directly before and after the fMRI session.
- **Images**: Immediately following the aftertaste induction in the scanner room, the participants were positioned in the scanner bore and the image presentation started. The participants viewed 30 images with sweet foods (e.g., cakes, ice cream), and 30 images with vegetables from a validated picture set (Blechert et al. 2014). Each image was presented for 2 s in an event-related design followed by an inter-stimulus-interval (fixation cross) ranging between 2 and 16 s. During the fMRI experiment, five pictures from both categories (sweets, vegetables) were rated concerning appetite (‘Please rate your appetite for the depicted food item’) and arousal (‘Please rate your excitement while looking at the depicted food item’) on 9-point Likert scales (1 = very low, 9 = very high).

### MRI recording and analysis

The MRI session was conducted with a 3 T scanner (Skyra, Siemens, Erlangen, Germany) with a 32-channel head coil. Functional runs were acquired using a T2*-weighted multiband EPI protocol (number of slices: 56, interleaved, flip angle = 72°, slice thickness: 3 mm; TE = 29.6 ms; TR = 2000 ms; multi-band accel. Factor = 2; FoV: 210 mm; in-plane resolution = 3 × 3 × 3 mm; total number of volumes: 315). The following parameters for the field map were used: number of slices: 56, interleaved, flip angle = 60°, slice thickness: 3 mm, TE 1 = 4.92, TE 2 = 7.38, TR = 594 ms, FoV: 210 mm; voxel size: 3 × 3 × 3 mm. Structural images were obtained using a T1-weighted MPRAGE sequence (voxel size: 0.9 × 0.9 × 0.9 mm; 192 transverse slices, FoV = 224 mm, slice thickness: 0.88 mm, TE = 1.89 ms, TR = 1680 ms; TI = 1000 ms, flip angle = 8°). All analyses were conducted using SPM12 (version: 6906; Wellcome Department of Cognitive Neurology, London). To account for saturation effects, the first three volumes of the functional runs were discarded. Susceptibility-induced distortions in brain images were corrected by FSL’s top-up. Three functional images acquired with negative blips were combined with three images with positive blips. The middle slice was used as a reference scan. Following slice timing, images were motion-corrected using realignment and unwarping. Subsequently, individual T1-weighted images were segmented into grey matter, white matter, and cerebrospinal fluid, and a skull-stripped image was created. Unwarped, slice-timed, and realigned images were matched to the skull-stripped image using the normalized mutual function. The obtained deformations fields were then used to bring functional images to MNI space. Finally, images were smoothed with a Gaussian kernel of 8 mm.

In the first level analyses, we compiled vectors for each event of interest in each session (Tea sweets, Tea vegetables, Water sweets, Water vegetables, rating scales) and entered them into the same design matrix to model event-related responses by the canonical hemodynamic response function together with time and dispersions derivatives. Data were high-pass filtered (128 s). The six movement parameters obtained during the realignment step were used as regressors of no interest. To reduce additional, unexplained noise in our data, a principal component analysis (PCA) was applied to extract individual time series from white matter (WM) and cerebrospinal fluid (CSF). Five PCAs of each tissue type (WM/CSF) were then introduced as covariates into the general linear model. PCA analyses were carried out with the CONN toolbox (version 18a; Whitfield-Gabrieli and Nieto-Castanon 2012). To reduce autocorrelations for adjacent time points, which are not part of task-related signals, the data were pre-whitened by using an AR(1) process.

### Statistical analyses

We computed an analysis of variance (ANOVA) to compare the hunger level at the beginning of the MRI session between Groups (high vs. low DP) and Conditions (tea vs. water). A mixed model ANOVA tested the effects of Group (high vs. low DP) and Time (before vs. after fMRI experiment) on reported disgust intensity and bitter intensity for the aftertaste. (The aftertaste ratings for water were 1 (the lowest possible value) and therefore not considered in the analysis).

An additional ANOVA was carried out to test the effects of Group (high vs. low DP), Condition (tea vs. water), and Picture Type (sweets vs. vegetables) on the picture ratings (arousal, appetite). Significant main effects and interactions were followed up by post-hoc t-tests.

For the fMRI data, we computed planned t-contrasts in the first-level analyses: Water sweets > Water vegetables;(Tea sweets > Tea vegetables) > (Water sweets > Water vegetables); (Water sweets > Water vegetables) > (Tea sweets > Tea vegetables)). Subsequently, in the second-level analyses, subject-specific contrast images were entered into a two-sample t-test to compare high vs. low disgust-prone participants. Exploratory whole-brain voxel intensity tests, as well as region of interest (ROI) analyses, were computed separately for the insula, operculum, OFC, mPFC (composite mask built from the superior frontal gyrus, anterior and para-cingulate cortex; Jahn et al. 2016), and basal ganglia based on previous studies on taste perception and disgust processing (Veldhuizen et al. 2011; Yeung et al. 2017; Schienle et al. 2002a). ROI masks were taken from the Harvard-Oxford cortical and subcortical structural atlases.

We also conducted exploratory generalized psychophysiological interaction (gPPI) analyses (McLaren et al. 2012) to investigate functional connectivity. PPI assesses the extent to which the experimental factor (contrast: Tea > Water: sweets > vegetables) modulates the connectivity of a specific brain region (‘seed’) with other regions, in terms of condition-specific covariation in residuals. Based on the fMRI activity findings, we selected the right mPFC mask as the seed region. Subject-specific contrast images were entered into a two-sample t-test to compare groups. The ROIs were the same as used for the BOLD contrast analysis.

For all analyses, results were small-volume corrected and considered significant if the peak-level statistic was below *p* < .05, corrected for family-wise error (FWE).

## Results

### Standardized taste test

The participants labeled all taste stimuli correctly. The computed t-tests revealed no group differences (high vs. low DP) in the intensity ratings (%) for the basic tastes sweet, sour, and salty. The high DP group rated the intensity of the bitter taste (quinine HCL) as marginally higher (*p* = .06) than the low DP group (see Table 1).

**Table 1:** Comparison of women with high vs. low disgust propensity (DP)

	Low_DP M (SD)	High_DP M (SD)	sig (2-tailed) p
Age (years)	24.43 (6.17)	24.23 (6.03)	.90
Years of education	12.69 (1.34)	12.79 (1.40)	.78
Taste Test Intensity [0–100%]			
NaCl	58.56 (26.59)	64.75 (25.38)	.36
Sucrose	41.81 (30.05)	40.56 (25.43)	.86
Citric acid	73.16 (24.02)	71.85 (24.99)	.84
Quinine HCL	65.47 (27.40)	78.92 (26.27)	.06
Appetite ratings for food pictures with bitter aftertaste [1..9]			
Sweets (water)	5.65 (1.81)	5.50 (2.06)	.76
Sweets (wormwood)	5.27 (1.88)	5.68 (2.05)	.42
Vegetables (water)	4.98 (1.83)	5.51 (1.90)	.27
Vegetables (wormwood)	5.43 (1.48)	5.18 (2.01)	.58
Arousal ratings for food pictures with bitter aftertaste [1..9]			
Sweets (water)	3.56 (1.47)	3.70 (2.04)	.77
Sweets (wormwood)	3.60 (1.49)	4.00 (1.58)	.32
Vegetables (water)	3.26 (1.51)	2.98 (1.45)	.46
Vegetables (wormwood)	3.55 (1.42)	3.43 (1.52)	.76

### Hunger level

The ANOVA showed no effects of Group (high vs. low DP) and Condition (tea vs. water) on the reported hunger level at the beginning of the MRI session (all *p* > .16). The average hunger level was medium (M = 4.7; SD = 2.1).

### Aftertaste ratings (wormwood)

#### Disgust intensity

The ANOVA revealed no significant effects (all *p* > .19). Directly after removing the tea from the mouth, the average disgust rating for the aftertaste was M = 6.88 (SD = 1.98). At the end of the experiment, the aftertaste rating was M = 6.53 (SD = 2.36).

#### Bitterness intensity

The effect of Group and the interaction Group x Time were not significant (*p* > .60). The main effect of Time reached statistical significance (F(1,58) = 14.3, *p* < .001). The mean rating for bitter intensity at the beginning of the experiment (directly after removing the tea from the mouth) was M = 8.10 (SD = 1.23) and at the end of the experiment M = 7.13 (SD = 1.84).

### Picture ratings

#### Arousal

There was a significant effect of Condition (F(1,58) = 12.45, *p* = .001) with higher arousal ratings in the wormwood condition (Table 1). The interaction Group x Condition (F(1,58) = 4.07, *p* = .048) resulted from higher arousal in the wormwood condition (difference tea minus water) reported by the DP_high group (M = 0.64, SD = 1.03) compared to the DP_low group (M = 0.17, SD = 0.73, t = 2.02, *p* = .048).

#### Appetite

The ANOVA for the appetite ratings revealed no significant effects (all *p* > .05).

In summary, the results for the aftertaste ratings were not in line with the hypothesis of a greater bitter sensitivity of the DP_high group compared to the DP_low group. Partially in line with the hypotheses, the DP_high group reported increased arousal (but not decreased appetite) for visual food cues that were coupled with a bitter aftertaste.

### Brain activity

#### Total-sample analysis

To obtain information about the general processing of the food images, we selected the water condition as a neutral context (without conflicting taste information) and analyzed the contrast Sweets <> Vegetables. The viewing of sweets compared to vegetables was associated with activation in the bilateral OFC (left: −18,15,−15, t = 3,52; *p* = .046; right: 21,9,−15, t = 3.98, *p* = .012) and basal ganglia (left: −24,6,−9, t = 4,23, *p* = .004; right: 21,9,−12, t = 4,41, *p* = .002; see supplementary Fig. S2). The reversed contrast indicated activation in the mPFC (peak: left superior frontal gyrus (x,y,z): −24,18,57, t = 3.90, *p* = .047). Additional total-sample contrasts are displayed in the supplementary Table S1.

#### Within-group analysis

The DP_high group was characterized by increased right mPFC activation (peak: anterior cingulate cortex) for the contrast ‘Tea > Water: Sweets > Vegetables’ (MNI coordinates (x,y,z): 12,42,0, t = 4.42; d = 1.65; p(FWE) = .037). The DP_low group displayed increased activity in the right insula (peak: ventral anterior insula; MNI coordinates (x,y,z): 30,18,−3; t = 3.47; d = 1.29; p(FWE) = .046) for the contrast ‘Water > Tea: Sweets > Vegetables’.

Exploratory correlation analysis demonstrated that within the DP_high group, difference score of arousal ratings (tea – water) for the food images (tea minus water) were positively associated with mPFC activity (x,y,z: 24, 6, 66, t = 4.56, p(FWE) = .030). The activation peak was located in the superior frontal gyrus.

#### Between-group analyses

**Fig. 1:**
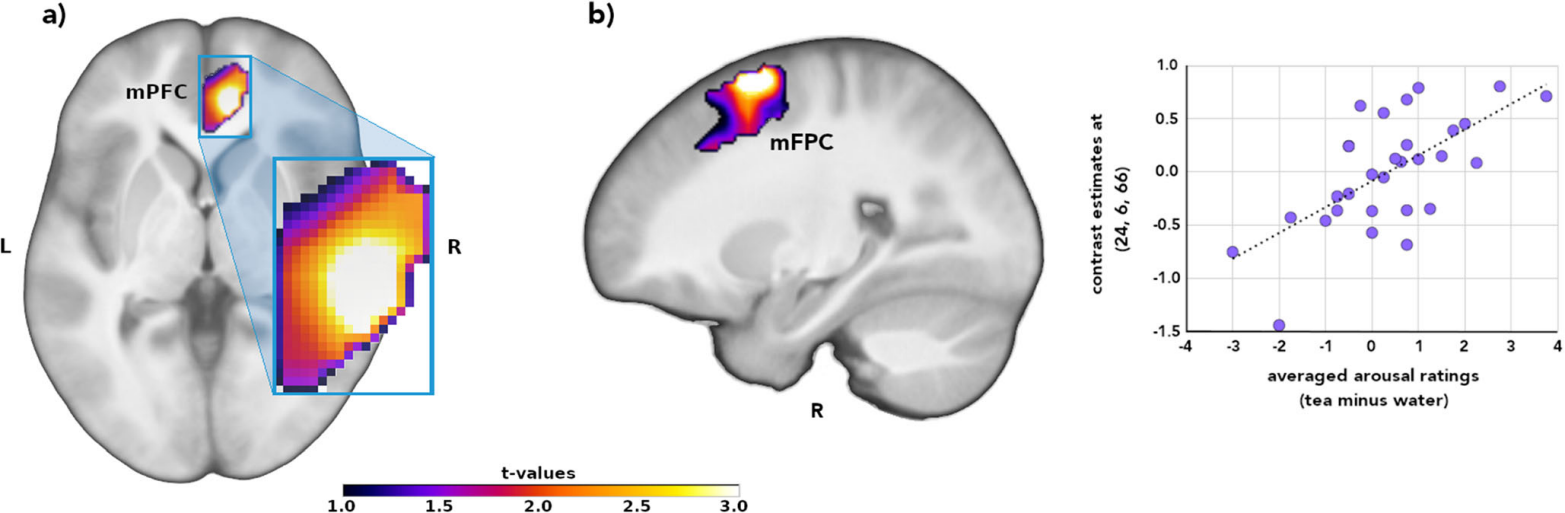
**a** Enhanced medial prefrontal cortex (mPFC; peak: anterior cingulate cortex) activity in disgust-prone women and **b** correlation between reported arousal and mPFC activity (contrast: Water - Tea: sweets - vegetables)

The group comparison indicated greater mPFC activation (peak: anterior cingulate cortex) for the DP_high group compared to the DP_low group for the contrast ‘Tea > Water: Sweets > Vegetables’ (x,y,z: 9,39,0, t = 4.06, p(FWE) = .038; d = 1.07; see Fig. 1).

#### Functional connectivity

The DP_high group showed increased functional connectivity of the right MPFC (peak: anterior cingulate cortex) with the right insula (peak: ventral anterior insula; MNI coordinates x,y,z: 39,0,−18, t = 3.26; d = 0.86; p(FWE) = .034) compared to the DP_low group (contrast; ‘Tea > Water: Sweets > Vegetables’).

In summary, the brain imaging results did not show the predicted group difference in insula activity during the experiment. However, disgust-prone women displayed increased ACC activity and increased mPFC-insula coupling during the cross-modal stimulation.

## Discussion

The present cross-modal fMRI study investigated how females high vs. low in disgust propensity (DP) process conflicting food-related information. The participants were presented with images depicting sweet foods and vegetables while they experienced either a disgusting or neutral aftertaste. The selected food images were perceived as appetizing, which was reflected by the ratings and the brain activity in the total sample.

In the water condition, the viewing of sweet foods compared to vegetables triggered increased activation in the basal ganglia and OFC. Both regions are involved in the decoding of the reward properties of stimuli, including the hedonic value of food (e.g. Blechert et al. 2016). According to evolutionary-based approaches, high-calorie (sweet) foods have a higher reward value than low-calorie foods such as vegetables (Schwab et al. 2017). Compared to sweets, images of vegetables were associated with stronger mPFC activation. The activation peak was located in the superior frontal gyrus (SFG). The SFG is critical for executive functions, such as the monitoring and mental manipulation of information. More specifically, this area has been reported to be involved in a variety of cognitive and motor control tasks (for a review see Li et al. 2013). For example, the SFG has a role in proactive control of impulsive responses, which also includes volitional appetite control (e.g., Tuulari et al. 2015). Vegetables are typically considered the less tasty but healthier food choice compared to sweets and therefore should be preferred.

The cross-modal stimulation (visual-gustatory) was associated with different activation patterns in the high vs. low DP group. Disgust-prone participants showed enhanced mPFC activity to images depicting sweets that were presented with a bitter aftertaste. The activation peak was in the anterior cingulate cortex (ACC). Additionally, the functional connectivity between the mPFC and the insula was enhanced in the DP_high group compared to the DP_low group.

The ACC has been associated with a variety of different functions, such as attention and conflict monitoring (for a summary see Eusten et al. 2012; Heekeren et al. 2008; Erickson et al. 2004). More specifically, the ACC (particularly the dorsal parts) compute prediction errors that reflect the deviation between expected and received outcomes (e.g., Alexander and Brown 2011; Eusten et al. 2012). Thus, the ACC encodes mismatch or conflict between incoming sensory input and also predicts adaptive responses, such as adaptive motor actions, autonomic and emotional responses (Alexander and Brown 2011).

In the present experiment, participants were exposed to pleasant visual food cues that were presented in an unpleasant (disgusting) gustatory context. Particularly concerning the sweet foods shown (e.g., cream cake, ice cream), the bitter taste can be considered as setting a conflicting and threatening context. Dairy products should never taste bitter because bitterness indicates possible spoilage (Glendinning 1994). Unsurprisingly, the DP_high group showed a stronger neural response to this type of conflict or cross-modal mismatch (visual cue of sweet food – bitter taste).

Contrary to our hypothesis, the DP_high group did not differ from the DP_low group in the sensory processing of the bitter stimulus. Both groups gave comparable ratings for the bitterness of the aftertaste. Moreover, both DP groups did not differ in their activation of the gustatory cortex (e.g., insula). Thus, DP was not associated with increased sensory bitter sensitivity. However, DP was associated with the appraisal of visual food cues presented in the context of the bitter taste. High disgust-prone females reported higher arousal for the food images in the bitter condition, which was positively correlated with activity in a prefrontal cognitive control area, the superior frontal gyrus (Li et al. 2013). This suggests a different integration and evaluation of the visual-gustatory information in the DP_high group compared to the DP_low group.

This interpretation is supported by the findings of the connectivity analysis. The DP_high group showed increased mPFC-insular connectivity when looking at sweet foods while having a bitter taste in the mouth. The mPFC is strongly interconnected with anterior insular areas, known to be involved in both interoception and taste perception (Eusten et al. 2012). Previous neurophysiological and brain imaging studies have shown that the fronto-insular complex (FIC) modulates arousal and plays a critical role in the awareness of changes in homeostatic states (e.g., Craig 2002). A bitter taste is a warning signal that something might be wrong with a specific food item. Additionally, the unusual combination of sensory characteristics of food (pleasant looks – disgusting taste) is a salient signal. According to Uddin (2015), the insula is the integral hub of the ‘salience network’ in the brain that assists target brain regions in the integration of stimulus information, as well as in the generation of appropriate behavioral responses. In this sense, the high DP group differed in cross-modal integration of disgust-relevant stimuli from the low DP group. This integration process is of great relevance according to the ‘disease avoidance mechanism’ of disgust (Curtis et al. 2011). To be able to avoid a certain harmful food in the future, integrated knowledge of its properties (e.g., looks beautiful, but is poisonous) is essential.

We need to mention the following limitations of the present investigation and implications for future research. We only studied females, due to their typically higher DP compared to males (e.g., Schienle et al. 2002b). Therefore, the results cannot be generalized to males. The two groups (DP_high, DP_low) did not differ in the perception of the wormwood tea, which was rated as extremely bitter and disgusting by all participants of the study. This very likely reflects a ceiling effect. When lower bitter concentrations were used as in the taste test with the quinine HCl solution, the DP_high group displayed greater bitter sensitivity than the DP_low group.

In the course of the experiment, there was a slight but statistically significant reduction of the reported bitter intensity for the wormwood aftertaste. On average, the average bitter rating decreased from M = 8.1 to 7.1 (9 = maximal value). In contrast, the disgust intensity did not change. Therefore, it seems unlikely that habituation had pronounced effects on the observed findings.

Finally, a future study should not only include cross-modal stimulation conditions (visual plus gustatory) but should also administer the gustatory and visual stimuli separately. These conditions could serve as a reference.

In conclusion, our findings indicate that the personality trait DP is associated with cross-modal integration processes of disgust-relevant information. Females high in DP were more alert to food-related sensory mismatch (pleasant visual features, aversive taste) than females low in DP.

## Supplementary Information

Figure S1 Bimodal distribution of the Questionnaire for the Assessment of Disgust Propensity (QADP) scores. Footnote: The sample showed a bimodal distribution of QADP scores. Therefore, we conducted a median split analysis and compared participants with high vs. low mean QADP scores. (PNG 38 kb)

Figure S2 Activation in the total sample for the contrast Water: Sweets – Vegetables (PNG 85 kb)

Table S1 (DOCX 17 kb)
