# Supplementary figures and images for: The processing of visual food cues during bitter aftertaste perception in females with high vs. low disgust propensity: an fMRI study
Source: Brain Imaging Behav. 2021 Feb 16;15(5):2532–9. doi: 10.1007/s11682-021-00455-2 (PMC8500869; doi:10.1007/s11682-021-00455-2)

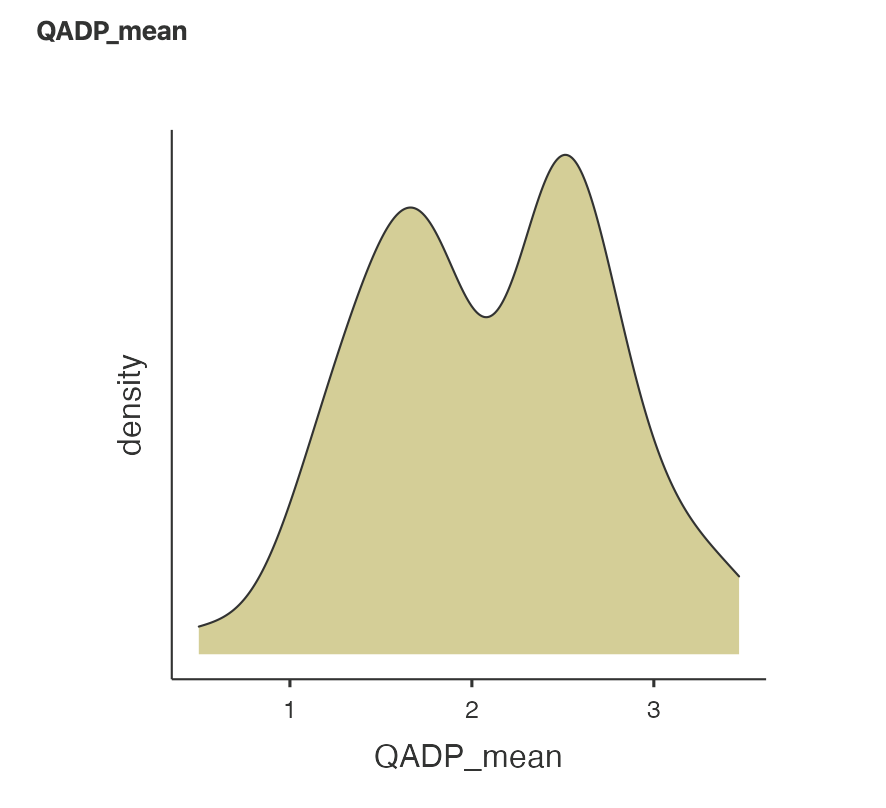

Supplement: Supplementary file 1 — Bimodal distribution of the Questionnaire for the Assessment of Disgust Propensity (QADP) scores. Footnote: The sample showed a bimodal distribution of QADP scores. Therefore, we conducted a median split analysis and compared participants with high vs. low mean QADP scores. (PNG 38 kb) [file 11682_2021_455_MOESM1_ESM.png]

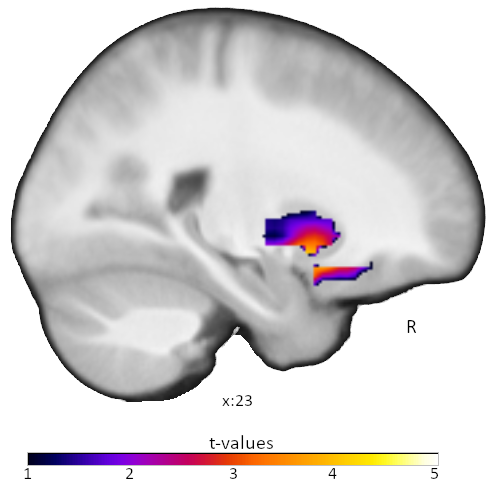

Supplement: Supplementary file 2 — Activation in the total sample for the contrast Water: Sweets – Vegetables (PNG 85 kb) [file 11682_2021_455_MOESM2_ESM.png]
