# Supplementary material for: The processing of visual food cues during bitter aftertaste perception in females with high vs. low disgust propensity: an fMRI study
Source: Brain Imaging Behav. 2021 Feb 16;15(5):2532–9. doi: 10.1007/s11682-021-00455-2 (PMC8500869; doi:10.1007/s11682-021-00455-2)
Supplement: Supplementary file 3 — (DOCX 17 kb) [file 11682_2021_455_MOESM3_ESM.docx]

**Supplementary Table 1: Activation in the total sample (n = 60) for the contrasts Water <> Tea, and Sweets <> Vegetables**

| **ROI** | **H** | **x** | **y** | **z** | **t-value** | **p(FWE)** |
| --- | --- | --- | --- | --- | --- | --- |
| **Water (Sweets + Vegetables) > Tea (Sweets + Vegetables)** | | | | | | |
| Insula | R | 39 | -3 | 3 | 3.88 | 0.014 |
| **Inferior temporal gyrus** | **L** | **-54** | **-60** | **-12** | **5.30** | **0.022** |
| **Fusiform gyrus** | **R** | **36** | **-33** | **-21** | **5.17** | **0.033** |
| **Sweets (Tea + Water) > Vegetables (Tea + Water)** | | | | | | |
| Caudate nucleus | L | -18 | 15 | -9 | 4.84 | 0.001 |
| OFC | L | -18 | 15 | -15 | 4.32 | 0.005 |
| OFC | R | 21 | 12 | -15 | 3.46 | 0.046 |
| **Inferior occipital gyrus** | **L** | **-48** | **-72** | **-9** | **14.01** | **<.001** |
| **Lateral occipital gyrus** | **L** | **-33** | **-87** | **12** | **10.11** | **<.001** |
| **Vegetables (Tea + Water) > Sweets (Tea + Water)** | | | | | | |
| mPFC (peak: superior frontal gyrus) | L | -18 | 25 | 45 | 4.64 | 0.005 |
| mPFC (peak: superior frontal gyrus) | R | 27 | 21 | 54 | 6.48 | <.001 |
| **Lingual gyrus** | **L** | **6** | **-84** | **-6** | **7.04** | **<.001** |
| ROI: region of interest; H: hemisphere (L: left, R: right); x,y,z: MNI coordinates; p(FWE): p-values corrected for family-wise error; OFC: orbitofrontal cortex; mPFC: medial prefrontal cortex; bold: whole-brain results | | | | | | |
